# Traditional Chinese Medicine Intervention Based on Metabolic–Epigenetic Axis: Mechanism and Treatment Strategy of Chronic Heart Failure

**DOI:** 10.3390/biom16070989

**Published:** 2026-07-06

**Authors:** Ji-Chao He, Jia-Ming Wei, Bin Wang, Ru-Fei Li, Wei Wang, Ya Li

**Affiliations:** 1 School of Pharmacy, Hunan University of Chinese Medicine, Changsha 410208, China; 2 Institute of Traditional Chinese Medicine Diagnostics, Hunan University of Chinese Medicine, Changsha 410208, China

**Keywords:** chronic heart failure, metabolic reprogramming, epigenetics, metabolites, traditional Chinese medicine

## Abstract

Chronic heart failure [CHF] is a progressive clinical syndrome characterized by structural and functional impairment of the myocardium, in which energy metabolic remodeling plays a central role. Increasing evidence suggests that metabolic disturbances in CHF are not only a consequence of reduced cardiac output but also active regulators of epigenetic remodeling, thereby contributing to disease progression. Key metabolites, including α-ketoglutarate, acetyl-CoA, NAD^+^, S-adenosylmethionine, succinate, and 2-hydroxyglutarate, influence the activity of DNA methyltransferases, histone-modifying enzymes, and other chromatin regulators, thereby linking metabolic status to transcriptional control. Through these mechanisms, metabolic abnormalities promote persistent activation of pathological gene programs associated with cardiomyocyte hypertrophy, fibrosis, inflammation, apoptosis, and mitochondrial dysfunction, forming a self-reinforcing metabolic–epigenetic feedback loop in CHF. Although current guideline-directed medical therapies improve symptoms and clinical outcomes, they do not directly target this metabolic–epigenetic axis. Traditional Chinese medicine (TCM), including bioactive compounds, herbal formulas, patent medicines, and injections, has demonstrated potential in preclinical studies to modulate myocardial energy metabolism, improve mitochondrial function, and influence epigenetic regulators such as SIRT1, AMPK, and TET/JmjC-dependent pathways. However, most available evidence is derived from experimental models, and causal relationships between metabolite regulation, epigenetic remodeling, and cardiac functional improvement remain insufficiently validated. This review summarizes current knowledge on metabolite-driven epigenetic regulation in CHF and evaluates emerging evidence on the role of TCM in modulating this network. We also critically discuss key limitations, including reliance on non-clinical models, incomplete pharmacokinetic understanding, and insufficient causal validation. Finally, we propose future directions based on multi-omics integration, single-cell and spatial technologies, and systems biology approaches to facilitate mechanistic clarification and translational development of metabolism-targeted strategies for CHF.

## 1. Introduction

Chronic heart failure (CHF) is a heterogeneous clinical syndrome characterized by structural and functional impairment of the myocardium. It represents the final common stage of many cardiovascular diseases and remains a major challenge in clinical practice and public health [1]. CHF is frequently associated with reduced exercise tolerance and poor quality of life [2], recurrent hospitalization and high mortality In recent years, the prevalence of CHF has continued to increase because of population aging [3] and the rising burden of risk factors such as hypertension, coronary artery disease, obesity and diabetes [4,5]. Current guideline-directed medical therapy (GDMT) for CHF mainly includes angiotensin-converting enzyme inhibitors, angiotensin receptor blockers, angiotensin receptor-neprilysin inhibitors, beta-blockers, mineralocorticoid receptor antagonists and sodium-glucose cotransporter 2 inhibitors [6]. These treatments can improve symptoms, reduce hospitalization and delay disease progression. However, they do not completely reverse the pathological remodeling process, and many patients still experience recurrent decompensation and poor long-term outcomes [7]. Therefore, it is necessary to further explore additional therapeutic targets that are closely related to the pathogenesis of CHF. The myocardium is a highly energy-demanding tissue that depends on mitochondrial oxidative phosphorylation to maintain normal contraction and relaxation [8]. During the development and progression of CHF, cardiomyocytes undergo profound metabolic remodeling, the healthy adult heart primarily relies on fatty acid β-oxidation for ATP production, whereas the failing heart gradually shifts toward a fetal-like metabolic pattern that depends more on glucose utilization and glycolysis, although this metabolic shift may provide short-term adaptation to hypoxia and stress, it eventually leads to inefficient ATP production, lactate accumulation, oxidative stress, mitochondrial dysfunction, cardiomyocyte apoptosis, hypertrophy and fibrosis [9]. Therefore, metabolic remodeling is not only a consequence of CHF but also an important driver of disease progression. The clinical importance of metabolic regulation in cardiovascular disease has also been highlighted by the rapid development of new therapies for metabolic disorders. Glucagon-like peptide-1 receptor agonists and dual glucose-dependent insulinotropic polypeptide/glucagon-like peptide-1 receptor agonists, such as semaglutide and tirzepatide, have recently attracted considerable attention because of their effects on body weight, glucose metabolism and cardiovascular outcomes [10]. Semaglutide has been approved to reduce the risk of major adverse cardiovascular events in adults with established cardiovascular disease and overweight or obesity, and tirzepatide has shown beneficial effects in patients with obesity-related heart failure with preserved ejection fraction [11,12]. These findings suggest that interventions targeting systemic metabolism may also influence cardiac structure, function and clinical outcomes. In addition, metabolic dysfunction-associated steatohepatitis [MASH] has emerged as an important metabolic disease closely related to obesity, insulin resistance, dyslipidemia and cardiovascular risk. Resmetirom, a liver-directed thyroid hormone receptor-β agonist, has been approved as the first pharmacological treatment for adults with non-cirrhotic steatohepatitis with moderate to advanced liver fibrosis. Although resmetirom mainly targets hepatic lipid metabolism and inflammation, its approval further emphasizes the growing importance of metabolic regulation in diseases that affect cardiovascular homeostasis [13]. These developments indicate that metabolism-centered therapeutic strategies are becoming increasingly relevant to cardiovascular medicine and provide a broader context for exploring metabolic interventions in CHF.

In recent years, CHF research has moved beyond the traditional view that metabolism only reflects changes in energy supply and demand; increasing evidence suggests that metabolic states can regulate gene expression through metabolite-mediated signaling [14]. Epigenetics refers to reversible mechanisms that regulate gene expression without altering the DNA sequence, including DNA methylation, histone modifications [15] and non-coding RNA-mediated regulation [16]. Key metabolites, such as α-ketoglutarate, acetyl-CoA [17], NAD^+^ [18] and methyl donor-related metabolites, can act as substrates, cofactors or inhibitors of epigenetic enzymes. By influencing DNA methylation and histone modification, these metabolites establish a close connection between metabolic remodeling and gene transcription. This concept provides a new perspective for understanding the persistent or memory-like pathological gene expression patterns observed in CHF.

Traditional Chinese medicine has a long history in the prevention and treatment of CHF [19,20]. Therapeutic principles such as supplementing qi, activating blood circulation, warming yang and promoting water metabolism have been widely used in clinical practice. Qili Qiangxin Capsule is a representative TCM compound formula with multiple pharmacological effects. Large-scale randomized controlled trials have shown that it can reduce heart failure hospitalization and cardiovascular mortality with good safety [21,22]. Modern pharmacological studies have also demonstrated that many active components of TCM, including ginsenosides and astragaloside IV, can improve myocardial energy metabolism, reduce oxidative stress and attenuate myocardial remodeling [23,24]. In addition to single active components, TCM compound formulas, Chinese patent medicines and TCM injections may regulate complex pathological networks through multi-component and multi-target synergistic effects. However, whether and how TCM corrects metabolite imbalance and thereby modulates epigenetic remodeling in CHF remain to be fully clarified. Therefore, this review summarizes the changes in key metabolites and their epigenetic regulatory mechanisms during metabolic reprogramming in CHF. It further discusses the potential regulatory effects of TCM active ingredients, compound formulas, patent medicines and injections on the metabolic-epigenetic axis. This review aims to provide a theoretical basis for clarifying the modern molecular mechanisms of TCM in CHF treatment and for developing new therapeutic strategies.

## 2. The Metabolic–Epigenetic Axis in Chronic Heart Failure

**Figure 1:**
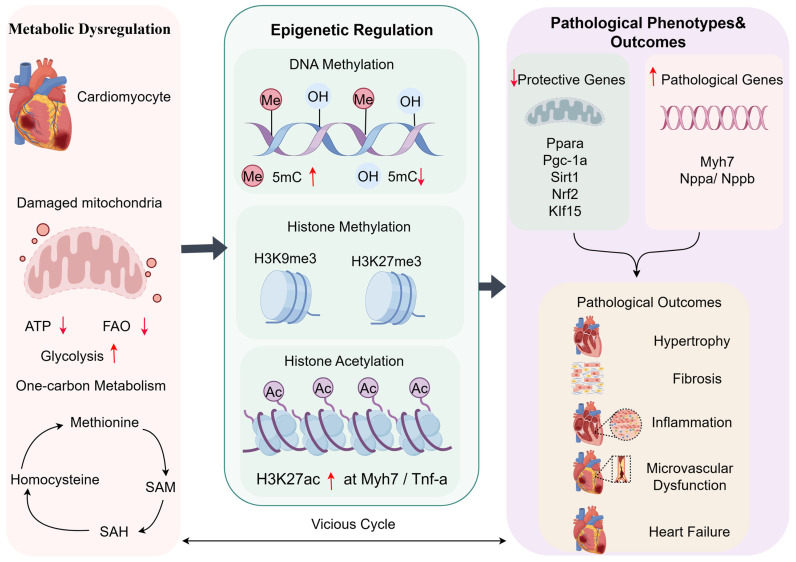
Metabolic–Epigenetic Dysregulation Drives a Vicious Cycle of Cardiac Pathology. This figure was drawn using the Figdraw platform [https://www.figdraw.com/, accessed on 4 July 2026], export ID: ITYOO42444.

In the setting of CHF, systemic and local myocardial metabolic disturbances create a unique metabolite milieu that, by influencing the activity of epigenetic enzymes, converts transient metabolic stress into persistent gene expression abnormalities, ultimately solidifying the pathological phenotype. In CHF, cardiac mitochondria serve as the “powerhouse” of cardiomyocytes. Under normal conditions, cardiac mitochondria generate the majority of ATP through oxidative phosphorylation to meet the substantial energy demands of continuous cardiac contraction, regulate Ca^2+^ homeostasis and apoptosis, and thereby maintain normal cardiomyocyte function and survival [25]; However, mitochondrial dysfunction in the failing heart leads to tricarboxylic acid (TCA) cycle disruption, an energy metabolism crisis, and, through its effects on one-carbon metabolism and the methionine cycle, an imbalance in methyl donor cycling. This results in abnormal abundance and ratios of key metabolites, including α-ketoglutarate (α-KG), acetyl-CoA (Ac-CoA), NAD^+^, S-adenosylmethionine (SAM)/S-adenosylhomocysteine (SAH), succinate, and 2-hydroxyglutarate (2-HG) [26,27]. These metabolites act as substrates, cofactors, or inhibitors of epigenetic enzymes and thereby provide a molecular link between metabolic stress and chromatin remodeling. Epigenetics refers to reversible and potentially heritable mechanisms that regulate gene expression without altering the DNA sequence. The major epigenetic mechanisms involved in cardiac remodeling include DNA methylation and histone modifications [15,16]. In CHF, these processes may contribute to the sustained activation of pathological gene programs related to cardiomyocyte hypertrophy, interstitial fibrosis, inflammation, apoptosis, and mitochondrial dysfunction [28]. However, it should be emphasized that many reported associations between metabolic intermediates and epigenetic remodeling in CHF remain correlative or are inferred from non-cardiac models. Therefore, the following section summarizes the better-established mechanisms. To provide an integrated overview of the metabolic–epigenetic axis in CHF, we constructed a conceptual framework shown in Figure 1.

### 2.1. Definitions of DNA Methylation and Histone Modifications

DNA methylation is one of the most conserved epigenetic modifications in mammalian cells. It mainly occurs at cytosine residues within CpG dinucleotides and is catalyzed by DNA methyltransferases (DNMTs). DNMTs transfer methyl groups from SAM to the fifth carbon of cytosine to generate 5-methylcytosine (5mC). DNMT1 primarily maintains pre-existing methylation patterns during DNA replication, whereas DNMT3A and DNMT3B mediate de novo methylation. In promoter CpG islands, DNA methylation generally suppresses gene transcription by directly interfering with transcription factor binding or by recruiting methyl-CpG-binding proteins and transcriptional corepressors; DNA demethylation is mainly mediated by the ten-eleven translocation(TET) family of dioxygenases. TET enzymes use Fe[II], oxygen, and α-KG as cofactors to oxidize 5mC into 5-hydroxymethylcytosine (5hmC), 5-formylcytosine, and 5-carboxylcytosine. These intermediates can be diluted through passive demethylation during DNA replication or removed through thymine DNA glycosylase-mediated base excision repair. Therefore, the balance between DNMT-mediated methylation and TET-mediated demethylation is closely linked to cellular metabolic status [29]. In CHF, metabolic imbalance regulates the activities of DNMTs and TET enzymes, leading to global hypermethylation and site-specific aberrant methylation. These changes silence genes involved in mitochondrial protection, energy metabolism, and antioxidant defense, while activating pathological gene programs associated with fetal gene re-expression, cardiomyocyte hypertrophy, and fibrosis [30]. Histone modifications are reversible covalent modifications of histone proteins, mainly occurring on the N-terminal tails of histones. These modifications include acetylation, methylation, phosphorylation, ubiquitination, and other post-translational marks. Among them, histone acetylation and histone methylation are particularly relevant to cardiac remodeling [31].

Histone acetylation is catalyzed by histone acetyltransferases (HATs), which use acetyl-CoA as the acetyl donor, whereas histone deacetylation is mediated by histone deacetylases (HDACs), including NAD^+^-dependent sirtuins. Histone acetylation is generally associated with chromatin relaxation and transcriptional activation [32]. Histone methylation is catalyzed by histone methyltransferases using SAM as the methyl donor, whereas histone demethylation is mediated in part by JmjC-domain histone demethylases, which require α-KG, Fe[II], and oxygen. The biological effects of histone methylation depend on the modified residue and the degree of methylation. For example, H3K4me3 is commonly associated with active promoters, whereas H3K9me3 and H3K27me3 are generally linked to transcriptional repression. Thus, histone modifications provide a dynamic and metabolite-sensitive regulatory layer that connects myocardial metabolic status with gene transcription [33].

### 2.2. α-KG Regulation of DNA and Histone Demethylation

α-KG is a key intermediate of the TCA that links carbon metabolism, amino acid metabolism, and mitochondrial redox homeostasis. It also serves as an essential co-substrate for the Fe^2+^/α-KG-dependent dioxygenase superfamily; this enzyme family includes TET DNA demethylases and JmjC domain-containing histone demethylases [34], which regulate DNA and histone demethylation, respectively. TET DNA demethylases catalyze the stepwise oxidation of 5mC to 5hmC, 5fC, and 5caC, whereas JmjC domain-containing enzymes catalyze the oxidative demethylation of methylated lysine residues on histones [26,34]. α-KG can translate cellular metabolic status into chromatin plasticity and transcriptional output, acting as an important bridge between myocardial metabolic remodeling and epigenetic reprogramming [35]. Recent studies have shown that dynamic changes in DNA methylation mediated by TET enzymes exert stage-specific effects on cardiac lineage commitment and cardiomyocyte formation during cardiomyocyte differentiation. In an adult mouse model of myocardial infarction, α-KG intervention was found to reduce H3K27me3 deposition at the promoter regions of cardiomyocyte cell-cycle-related genes and promote the transcriptional activation of these genes. Moreover, when JMJD3 was inhibited or knocked down, the α-KG-induced reduction in H3K27me3, activation of cell-cycle genes, and cardiomyocyte proliferative effects were all attenuated. α-KG can relieve the epigenetic repression of cardiomyocyte cell-cycle genes by activating JMJD3-dependent H3K27me3 demethylation, thereby promoting adult cardiomyocytes to re-enter the proliferative program and improving cardiac regeneration after myocardial infarction [33,36]. In CHF, cardiomyocytes are chronically exposed to mitochondrial dysfunction, oxidative stress, and energy metabolic remodeling. Reduced TCA flux, together with impairment of IDH2- and α-KGDH/NNT-related metabolic pathways, may decrease α-KG production and weaken NADPH-dependent antioxidant defense [37]. These epigenetic abnormalities may silence genes involved in mitochondrial biogenesis, fatty acid oxidation, antioxidant defense, and cardiomyocyte maturation, including Ppargc1a and Tfam, thereby aggravating myocardial energy metabolic disorder, mitochondrial injury, and pathological remodeling [38,39]. In a mouse model of pressure overload-induced heart failure, α-KG administration was found to restore NAD^+^/SIRT1 signaling, improve mitophagy, inhibit ferroptosis, and significantly attenuate myocardial hypertrophy and fibrosis. These findings suggest that α-KG deficiency may suppress the NAD^+^/SIRT axis, thereby promoting the sustained activation of gene programs associated with hypertrophy, inflammation, and fibrosis [40]. Therefore, α-KG represents a critical regulatory node in the vicious cycle of “metabolic abnormality–epigenetic imbalance–pathological gene activation” in chronic heart failure.

### 2.3. Acetyl-CoA and NAD^+^ in Histone Acetylation Homeostasis

Acetyl-CoA [Ac-CoA] is the principal acetyl-group donor for histone acetyltransferases(HATs), thereby linking cellular metabolic state to histone acetylation and chromatin accessibility. In CHF, impaired mitochondrial oxidative metabolism and altered substrate utilization may disturb Ac-CoA generation and subcellular availability, which could contribute to maladaptive histone acetylation remodeling and pathological gene expression [41,42]. Insufficient Ac-CoA availability may further exacerbate mitochondrial dysfunction, leading to reduced α-KG synthesis, disruption of the methionine cycle, and diminished activities of TET enzymes and JmjC domain-containing histone demethylases. Collectively, these alterations contribute to DNA hypermethylation and aberrant histone methylation [43]. Despite the overall reduction in cellular Ac-CoA availability, aberrantly enhanced ATP-citrate lyase (ACLY)-mediated Ac-CoA production within the nucleus may cause compartment-specific epigenetic dysregulation. Increased nuclear Ac-CoA promotes histone acetylation at profibrotic gene loci and may facilitate the recruitment of DNA methyltransferases (DNMTs), leading to promoter hypermethylation and transcriptional silencing of protective genes, including genes involved in mitochondrial homeostasis [44]. Under physiological conditions, HATs use Ac-CoA as a substrate to transfer acetyl groups to lysine residues on histones, thereby relaxing chromatin structure and activating genes involved in myocardial energy metabolism and antioxidant defense. In CHF, reduced Ac-CoA availability restricts HAT catalytic activity and decreases global histone acetylation. However, this global reduction is accompanied by locus-specific acetylation abnormalities, including aberrant hyperacetylation at prohypertrophic and proinflammatory gene loci, such as Myh7 and TNF-α, and hypoacetylation at loci associated with mitochondrial protection and antioxidant defense, such as Tfam. Together, these epigenetic alterations promote cardiac hypertrophy, inflammation, and fibrosis [45,46]. Furthermore, enhanced nuclear ACLY activity can selectively increase histone H3 lysine 27 acetylation (H3K27ac), activate profibrotic gene expression, promote the differentiation of cardiac fibroblasts into myofibroblasts, and ultimately accelerate the deterioration of cardiac function [47].

Nicotinamide adenine dinucleotide [NAD^+^] is a central coenzyme linking energy metabolism to epigenetic regulation. It serves not only as a key electron carrier in metabolic processes such as glycolysis and oxidative phosphorylation but also as an essential cofactor for the sirtuin (SIRT) family deacetylases. Therefore, cellular NAD^+^ homeostasis directly influences the metabolic–epigenetic–pathological gene regulatory network in CHF, serving as a critical hub for maintaining myocardial energy metabolism and epigenetic homeostasis [48]. NAD^+^ is an essential cosubstrate for the SIRT family of class III histone deacetylases, which, together with acetyl-CoA-dependent histone acetyltransferases, maintains the dynamic balance between histone acetylation and deacetylation [49]. In the nucleus, SIRT1 deacetylates histone and non-histone substrates, including H3K9ac, thereby suppressing proinflammatory and prohypertrophic signaling [50]. In mitochondria, SIRT3 regulates the NAD^+^-dependent deacetylation of multiple metabolic enzymes, thereby preserving mitochondrial function and myocardial energy homeostasis [51]. NAD^+^ depletion in heart failure markedly reduces SIRT1 and SIRT3 activities and impairs histone and protein deacetylation. This disruption compromises the coordinated regulation of acetylation by NAD^+^-dependent deacetylases and Ac-CoA-dependent acetyltransferases, resulting in aberrant acetylation at pathological gene loci, including Myh7 and TNF-α. Concurrently, dysregulated acetylation at protective gene loci, such as Tfam, may impair mitochondrial homeostasis and further aggravate myocardial metabolic dysfunction, oxidative stress, and apoptosis. In addition, SIRT1 can indirectly regulate the specificity of histone acetylation by deacetylating acetylation-associated enzymes, including histone acetyltransferases and histone deacetylases. Therefore, reduced SIRT1 activity may further disrupt cellular acetylation homeostasis [52,53].

### 2.4. SAM/SAH Ratio and Methylation Potential

S-adenosylmethionine [SAM] and S-adenosylhomocysteine [SAH] constitute a central metabolic pair that regulates cellular methylation capacity. SAM serves as the principal methyl donor for a broad range of methyltransferases, including DNA methyltransferases (DNMTs) and histone methyltransferases [54]. Under physiological conditions, SAM-dependent methylation contributes to the maintenance of a dynamic balance between gene repression and activation through locus-specific DNA and histone methylation. SAH is generated as a by-product of SAM-dependent methylation reactions and acts as a potent feedback inhibitor of multiple methyltransferases. Accordingly, the SAM/SAH ratio, often referred to as the methylation index or methylation potential, reflects both the availability of methyl donors and the cellular capacity to sustain methylation reactions. It therefore represents a critical metabolic link among one-carbon metabolism, the methionine cycle, and epigenetic regulation. Disruption of SAM/SAH homeostasis may consequently play a central role in the metabolic and epigenetic abnormalities associated with CHF [55]. In CHF, mitochondrial dysfunction, oxidative stress, inflammation, and alterations in energy-substrate utilization collectively disrupt one-carbon metabolism and the methionine cycle, thereby limiting SAM synthesis and regeneration. Reduced SAM availability restricts methyl-group transfer by DNMTs and histone methyltransferases. At the same time, the accumulation of SAH and homocysteine-related metabolites may further impair methylation reactions because elevated SAH competitively inhibits methyltransferase activity. These metabolic disturbances can induce global and locus-specific abnormalities in DNA and histone methylation. Consequently, the transcription of genes involved in mitochondrial biogenesis and fatty acid oxidation, including Ppargc1a, Tfam, and their downstream pathways, may be repressed. In contrast, fetal cardiac stress-response genes, such as Nppa and Nppb, may be reactivated through the combined effects of altered DNA methylation, histone modifications, and chromatin accessibility, thereby stabilizing the pathological phenotype of heart failure [56]. Efficient hydrolysis and clearance of SAH are essential for maintaining methylation reactions. Abnormal SAH accumulation can suppress methyltransferase activity even when intracellular SAM levels are not markedly reduced. This inhibition may result in aberrant remodeling of histone methylation at multiple lysine residues, including H3K4, H3K9, H3K27, and H3K36 [57,58]. Disruption of the SAM/SAH ratio may also indirectly affect the balance between protein acetylation and deacetylation through metabolic crosstalk. SAM deficiency-associated methylation abnormalities may alter the expression of acetylation-regulating enzymes, including histone acetyltransferases and histone deacetylases. In addition, SAH accumulation and the associated oxidative stress may further impair mitochondrial function, reduce acetyl-CoA production, and deplete NAD^+^. These changes may limit substrate availability for histone acetyltransferases and inhibit the activity of NAD^+^-dependent sirtuin deacetylases, ultimately disrupting acetylation–deacetylation homeostasis [59].

### 2.5. Succinate and 2-Hydroxyglutarate as Inhibitors of α-KG-Dependent Enzyme

Succinate and 2-hydroxyglutarate (2-HG) are not only metabolites associated with the TCA but also important signaling molecules that link metabolic disturbances to epigenetic dysregulation. Owing to their structural similarity to α-KG, both metabolites can act as competitive antagonists of α-KG and inhibit Fe^2+^/α-KG-dependent dioxygenases, including JmjC domain-containing histone demethylases and TET DNA demethylases. Consequently, abnormal accumulation of succinate or 2-HG impairs JmjC-mediated histone demethylation and induces aberrant remodeling of histone methylation at sites such as H3K9me3, H3K27me3, and H3K36me2/3, thereby altering chromatin accessibility and transcriptional programs [60]. During the progression of CHF, myocardial energy metabolism gradually shifts from fatty acid oxidation toward greater reliance on glycolysis, accompanied by impaired mitochondrial oxidative phosphorylation and an imbalance in the abundance of TCA-cycle intermediates. Dysfunction of succinate dehydrogenase (SDH) can disrupt the balance between myocardial fatty acid oxidation and glycolysis and has been associated with dilated cardiomyopathy and heart failure phenotypes [61]. By competitively inhibiting α-KG-dependent dioxygenases, succinate and 2-HG can impair JmjC domain-containing histone demethylases and thereby promote abnormal accumulation of histone methylation marks [62]. Although much of this mechanism has been established in non-cardiac systems, recent cardiac evidence indicates that SDH deficiency and succinate accumulation can induce epigenetic remodeling, suppress FAO-related gene expression, and reinforce the fetal-like metabolic phenotype of the failing heart [63].

## 3. Regulation of the Metabolism-Epigenetic Axis by Traditional Chinese Medicine

### 3.1. Active Ingredients of Traditional Chinese Medicine

Active components of traditional Chinese medicine can intervene in the pathological progression of CHF at multiple levels by regulating the metabolism–epigenetics axis. The core mechanism lies in their precise action on the “convergence points” between metabolic processes and epigenetic modification systems. By improving mitochondrial function and the status of the tricarboxylic acid cycle, these components regulate the dynamic balance between α-KG and its competitive inhibitors, thereby influencing the activity of epigenetic enzymes dependent on these metabolic intermediates. Meanwhile, active components of TCM can also increase intracellular NAD^+^ levels, restore SIRT deacetylase activity, and improve histone acetylation homeostasis [64]. In addition, by improving the methyl donor cycle and suppressing inflammatory signaling, they help maintain DNA and histone methylation homeostasis and reduce epigenetic abnormalities induced by upstream inflammatory stimuli [65]. These synergistic effects not only improve myocardial energy metabolism and mitochondrial function but also alleviate myocardial inflammation and fibrosis, thereby promoting the recovery of cardiac structure and function.

By improving mitochondrial function and the status of the tricarboxylic acid cycle, these components regulate the dynamic balance between α-KG and its competitive inhibitors, thereby influencing the activity of epigenetic enzymes dependent on these metabolic intermediates. Meanwhile, active components of TCM can also increase intracellular NAD^+^ levels, restore SIRT deacetylase activity, and improve histone acetylation homeostasis [64]. In addition, by improving the methyl donor cycle and suppressing inflammatory signaling, they help maintain DNA and histone methylation homeostasis and reduce epigenetic abnormalities induced by upstream inflammatory stimuli [65]. These synergistic effects not only improve myocardial energy metabolism and mitochondrial function but also alleviate myocardial inflammation and fibrosis, thereby promoting the recovery of cardiac structure and function.

Salvianolic acid B mainly maintains energy metabolism stability through antioxidant and mitochondrial protective effects. It may improve the ratio between α-KG and metabolic inhibitors such as succinate, optimize the catalytic environment of JmjC demethylases, and thereby indirectly promote the restoration of histone demethylation modifications. The combined application of ginsenoside Rg1 and salvianolic acid B may further improve myocardial mitochondrial function and energy metabolism, correct DNA hypermethylation and abnormal histone methylation caused by metabolic imbalance, and reduce cardiac remodeling, myocardial hypertrophy, and functional impairment by suppressing the expression of genes related to fibrosis and myocardial hypertrophy [66,67]. Their combination also exhibits more pronounced cardioprotective effects, including reducing myocardial infarct size, improving cardiac structure and function, and suppressing the release of inflammatory factors [68,69], thereby reflecting the multi-target and multi-level systemic regulatory potential of active components of TCM. Nevertheless, direct evidence regarding the regulation of JmjC histone demethylases by salvianolic acid B remains relatively insufficient, and its effects are largely inferred from changes in metabolites and mitochondrial protection. Therefore, future studies should include detection of histone methylation markers, such as H3K4me3, H3K9me3, and H3K27me3, combined with assays of JmjC demethylase activity and ChIP-qPCR/ChIP-seq analyses, to determine whether salvianolic acid B can restore the histone methylation status at specific pathological gene loci. At the same time, JmjC inhibitors, knockdown of key demethylases, or overexpression experiments could be used to further determine whether the effects of salvianolic acid B on myocardial hypertrophy and fibrosis depend on histone demethylation mechanisms. For the combined effects of Rg1 and salvianolic acid B, additional evaluation of drug synergy should be performed, such as combination index analysis, dose–response analysis, and multi-omics comparisons among single-treatment and combination-treatment groups, in order to distinguish whether their effects are merely additive or represent true synergistic regulation.

NAD^+^ depletion is another key event in the dysregulation of the metabolism–epigenetics axis in CHF. As an essential coenzyme for the class III histone deacetylase SIRT family, reduced NAD^+^ levels impair SIRT1- mediated deacetylation and SIRT3-mediated deacetylation, thereby aggravating mitochondrial dysfunction, oxidative stress, and cardiomyocyte apoptosis, and promoting the progression of CHF [70]. Supplementation with NAD^+^ precursors, such as β-nicotinamide mononucleotide (NMN) or nicotinamide riboside(NR), can restore intracellular NAD^+^ levels in cardiomyocytes and reactivate SIRT1 and SIRT3. Therefore, this represents a relatively well-supported and mechanistically defined intervention target [71,72]. Resveratrol can upregulate the expression of nicotinamide phosphoribosyltransferase, enhance the NAD^+^ salvage synthesis pathway, and expand the intracellular NAD^+^ pool in cardiomyocytes. Meanwhile, as a natural allosteric activator of SIRT1, resveratrol can enhance SIRT1 enzymatic activity in the presence of NAD^+^, promoting the deacetylation of histones such as H3K9 and H3K56, as well as key non-histone substrates including PGC-1α, FOXO1, and p53. Through these mechanisms, it drives mitochondrial biogenesis, enhances antioxidant gene expression, suppresses pro-apoptotic signaling, and exerts multiple cardioprotective effects [73,74,75]. Although the regulatory role of resveratrol in the NAD^+^–SIRT1 pathway has a relatively strong research foundation, whether it regulates myocardial remodeling-related genes through specific histone deacetylation sites in CHF models remains to be further clarified. Future studies should measure the NAD^+^/NADH ratio, NAMPT expression, SIRT1/SIRT3 activity, and histone acetylation levels such as H3K9ac and H3K56ac. In parallel, SIRT1 or SIRT3 inhibitors, as well as gene knockdown or knockout models, should be used to verify whether the cardioprotective effects of resveratrol depend on the NAD^+^-SIRT axis. In addition, the acetylation status of non-histone substrates such as PGC-1α, FOXO1, and p53 could be further analyzed, together with mitochondrial respiratory function, ROS levels, apoptosis, and cardiac function indicators, to establish a complete mechanistic chain of “resveratrol–NAD^+^ salvage synthesis–SIRT deacetylation–mitochondrial protection–heart failure improvement”.

### 3.2. Chinese Medicine Compound and Chinese Patent Medicine

Unlike active compounds that target a single molecular site, traditional Chinese medicine formulas are characterized by multiple components and multiple targets. This enables them to act simultaneously on several nodes of the metabolism–epigenetics axis and to regulate complex biological networks through additive or synergistic effects, thereby conferring an advantage in holistic intervention. Qiliqiangxin Capsule, a Chinese patent medicine used for the treatment of CHF, provides a representative example. It consists of multiple medicinal herbs, including *Astragalus membranaceus*, *Panax ginseng*, *Aconitum carmichaelii*, *Salvia miltiorrhiza*, *Lepidium apetalum*, *Alisma plantago-aquatica*, *Polygonatum odoratum*, *Cinnamomum cassia*, *Carthamus tinctorius*, *Periploca sepium*, and *Citrus reticulata*, and is traditionally used to tonify Qi and warm Yang, promote blood circulation and unblock the vessels, and induce diuresis to alleviate edema. As the principal herbs in the formula, *Astragalus membranaceus* and *Panax ginseng* contribute key bioactive constituents, including astragaloside IV and ginsenosides such as Rg1, Rc, and Rb1, which may synergistically act on cellular energy-sensing and metabolic regulatory pathways, particularly the AMPK/SIRT1/PGC-1α and PGC-1α/PPARα axes. Previous experimental studies have demonstrated that astragaloside IV improves myocardial hypertrophy by modulating PGC-1α-associated bioenergetic programs and may attenuate myocardial fibrosis via SIRT1/PGC-1α-related signaling [76]. Ginsenoside Rg1 has been reported to activate AMPK- or SIRT1-dependent pathways, thereby enhancing mitochondrial function and ameliorating adverse cardiac remodeling [77]. Ginsenoside Rc functions as a SIRT1 activator and promotes PGC-1α-mediated mitochondrial biogenesis and myocardial energy metabolism [78]. Ginsenoside Rb1 has been shown to improve heart failure–associated energetic impairment and myocardial injury through the ROS/PPARα/PGC-1α signaling pathway [79]. Collectively, these bioactive constituents may enhance myocardial energy metabolism and increase NAD^+^ biosynthesis and availability, thereby providing a metabolic basis for restoring Sirtuin family deacetylase activity and potentially contributing to the correction of aberrant histone acetylation. *Salvia miltiorrhiza* and Carthamus tinctorius, representative blood-activating and stasis-resolving herbs, primarily exert cardioprotective effects through the restoration of redox homeostasis and preservation of mitochondrial integrity [80]. Salvianolic acid B, a major active constituent of *Salvia miltiorrhiza*, exhibits potent antioxidant and anti-apoptotic activities by scavenging excessive reactive oxygen species (ROS), thereby maintaining mitochondrial membrane integrity, preserving electron transport chain function, and sustaining physiological TCA activity. Improvement of mitochondrial function further contributes to the maintenance of α-KG homeostasis and reduces the accumulation of oncometabolites such as succinate, thereby supporting the activity of α-KG-dependent epigenetic enzymes, including TET DNA demethylases and JmjC histone demethylases [81,82]. This facilitates DNA demethylation and restoration of physiological epigenetic regulation. In parallel, attenuation of oxidative stress reduces DNA damage burden and limits excessive consumption of NAD^+^ during DNA repair processes, thereby indirectly preserving the activity of NAD^+^-dependent Sirtuin deacetylases [83]. Collectively, these findings suggest that *Salvia miltiorrhiza* and related herbs establish a favorable metabolic microenvironment for epigenetic restoration in cardiomyocytes through mitochondrial protection and metabolite homeostasis regulation. The “warming and circulation-promoting” herb pair composed of *Aconitum carmichaelii* and *Cinnamomum cassia* primarily improves myocardial contractility and systemic perfusion. Studies have shown that, in *Aconitum*, aconite alkaloids enhance cardiac contractility within a safe pharmacological window by modulating adrenergic receptor signaling. Meanwhile, cinnamaldehyde derived from *Cinnamomum cassia* exerts vasodilatory effects, thereby improving peripheral circulation and tissue perfusion [84,85,86]. Their synergistic action increases cardiac output, enhances oxygen and nutrient delivery to the myocardium, and facilitates metabolic waste clearance, thereby providing a favorable physiological basis for metabolic recovery and epigenetic stability in cardiomyocytes. Furthermore, CHF is characterized by fluid retention, tissue edema, and systemic metabolic dysregulation, all of which exacerbate myocardial metabolic stress. Herbal components such as *Descurainia sophia*, *Alisma orientale*, and *Periploca sepium* alleviate volume overload through multi-target mechanisms. Cardiac glycosides from *Descurainia sophia* exert positive inotropic effects [87], while *Alisma orientale* extracts regulate aquaporin expression to promote diuresis and relieve sodium-water retention [88]. In addition, hesperidin derived from Citrus reticulata exhibits anti-inflammatory, antioxidant, and anti-edema properties via suppression of the TNF-α/NF-κB signaling pathway, thereby reducing inflammatory infiltration [89,90,91]. *Polygonatum odoratum* polysaccharides further improve insulin resistance and lipid metabolic disorders, contributing to systemic metabolic rebalancing [92]. From a metabolism–epigenetics perspective, Qiliqiangxin Capsule may enhance myocardial NAD^+^ and α-KG bioavailability through multi-component synergistic regulation, thereby activating SIRT1-dependent deacetylation and supporting TET-mediated DNA demethylation [40]. At the epigenetic level, this may contribute to the repression of fetal gene reprogramming markers such as ANP, BNP, and β-MHC via histone deacetylation and DNA methylation remodeling, while simultaneously promoting the expression of genes associated with mitochondrial biogenesis and antioxidant defense [93]. Consequently, Qiliqiangxin Capsule may exert therapeutic effects through a continuous regulatory axis involving correction of metabolic disturbance, epigenetic landscape remodeling, reversal of pathological gene programs, and improvement of cardiac function. However, the aforementioned mechanistic model, ranging from molecular networks to holistic therapeutic effects, still requires further validation and refinement through integrated multi-omics approaches. The case of Qiliqiangxin Capsule illustrates that TCM formulas can exert therapeutic effects through multi-target and multi-level regulatory mechanisms, thereby disrupting the vicious cycle of “metabolic dysregulation–epigenetic disturbance–pathological phenotype fixation” in heart failure. This also reflects the research value of the holistic therapeutic philosophy of TCM within the modern framework of systems biology. In addition to Qiliqiangxin Capsule, a variety of clinically commonly used TCM formulas similarly follow the principle of “holistic regulation and multi-target synergy.” These formulations modulate the metabolism–epigenetics axis at multiple levels, including energy substrate metabolism, redox homeostasis, water metabolism, and inflammatory microenvironment regulation. The core effects and underlying mechanisms are summarized in Table 1.

### 3.3. Traditional Chinese Medicine Injections

Traditional Chinese medicine injections are important dosage forms developed during the modernization of TCM. They are characterized by relatively rapid onset, high bioavailability, and multi-component synergistic effects, and have been widely used in the clinical treatment of CHF [105]. Compared with oral herbal formulas and Chinese patent medicines, traditional Chinese medicine injections can rapidly enter the systemic circulation after intravenous administration and exert more direct regulatory effects on myocardial energy metabolism, oxidative stress, inflammatory responses, and mitochondrial function [106,107]. Although commonly used injections for CHF differ in botanical origin, core constituents, and pharmacological material basis, their actions largely converge on improving myocardial energy metabolism and restoring mitochondrial function [106]. By regulating signaling pathways such as PPARα/SIRT1/PGC-1α, AMPK/SIRT, PI3K/Akt, and Nrf2/HO-1, these injections may stabilize key metabolite pools, including NAD^+^, α-ketoglutarate, acetyl-CoA, and SAM, thereby influencing epigenetic processes such as DNA methylation, histone acetylation, and histone methylation [28,108]. Through these mechanisms, they participate in the regulation of pathological processes including myocardial hypertrophy, inflammation, apoptosis, oxidative stress, and myocardial fibrosis, ultimately protecting cardiac function and delaying ventricular remodeling. The core effects, major mechanisms, and potential epigenetic regulatory features of these injections are summarized in Table 2.

Shenmai Injection (SMI) is one of the representative traditional Chinese medicine injections used for the treatment of heart failure. It is mainly derived from *Panax ginseng* and *Ophiopogon japonicus*, and its pharmacologically active components include ginsenosides, ophiopogonins, and polysaccharides. Studies have shown that SMI can regulate lipid metabolism and mitochondrial function, thereby improving post-myocardial infarction heart failure. Based on network pharmacology and animal experiments, SMI has been found to improve cardiac function, attenuate myocardial injury, and enhance myocardial lipid metabolism in rats with heart failure after myocardial infarction by upregulating the PPARα/SIRT1/PGC-1α signaling pathway [108]. SMI also promoted the recovery of fatty acid oxidation-related pathways, improved insufficient myocardial lipid substrate utilization under heart failure conditions, and partially reversed pathological metabolic reprogramming characterized by impaired fatty acid oxidation and increased dependence on glycolysis [108]. These findings are closely aligned with the core concept of the metabolism–epigenetics axis. PPARα is a key transcription factor regulating myocardial fatty acid uptake and β-oxidation, whereas PGC-1α is a critical coactivator involved in mitochondrial biogenesis and oxidative phosphorylation [109]. Activation of PPARα/PGC-1α-related signaling by SMI can promote mitochondrial oxidative metabolism and fatty acid oxidation, improve ATP deficiency, and restore impaired tricarboxylic acid cycle function [38]. Recovery of tricarboxylic acid cycle flux helps maintain the homeostasis of metabolic intermediates such as α-ketoglutarate, thereby providing a more favorable metabolic environment for TET DNA demethylases and JmjC domain-containing histone demethylases. Accordingly, the improvement of energy metabolism induced by SMI may alleviate abnormal DNA and histone methylation caused by α-ketoglutarate deficiency and the accumulation of competitive inhibitors such as succinate in heart failure. SIRT1 is an NAD^+^-dependent deacetylase and serves as an important node connecting energy metabolism with epigenetic regulation [49]. In heart failure, NAD^+^ depletion and mitochondrial dysfunction weaken SIRT1/SIRT3-mediated deacetylation, thereby aggravating the abnormal activation of gene programs associated with inflammation, hypertrophy, apoptosis, and mitochondrial injury [110]. By upregulating the SIRT1/PGC-1α pathway, SMI may enhance mitochondrial biogenesis and fatty acid oxidation through deacetylation of PGC-1α, thereby improving myocardial energy metabolism [111]. It may also restore SIRT1-related deacetylase activity, inhibit inflammatory and apoptotic signaling pathways such as NF-κB and p53, reduce the expression of inflammatory cytokines including TNF-α and IL-6, and alleviate cardiomyocyte injury and interstitial remodeling [112]. Since SIRT1 is also involved in histone deacetylation, activation of this pathway provides a clear metabolism–epigenetics link through which SMI may regulate histone acetylation homeostasis.

**Table 2:** Summary of the core effects and mechanisms of TCM injections in improving chronic heart failure by regulating the metabolic–epigenetic axis.

Name	Pathway	Metabolites	Epigenetic Effects	Improved Heart Function
Shenmai injection [111]	PPARα SIRT1 PGC-1α	NAD^+^ α-KG succinate ATP	Activates SIRT1-related deacetylation	improve myocardial tissue structure disorder
Danhong injection [113]	miR-126 ERK VEGF	Salvianolic acid A, B, C Hydroxysafflor yellow A	Upregulate miR-126 and activate ERK phosphorylation	Inhibit myocardial infarction
Guanxinning injection [114]	MMP1 SLC7A11 GPX4	GSH GPX4 ROS	Reduce oxidative stress-driven epigenetic remodeling and inhibit profibrotic gene activation	inhibit cardiac fibrosis and hypertrophy, improve cardiac remodeling in heart failure
Shenfu injection [115]	HIF-1α AMPK SIRT NF-κB	ATP Lactate TMAO ROS NAD^+^	Restore NAD^+^-dependent SIRT activity and reduce inflammation-mediated epigenetic abnormalities	Improve cardiac contractile function, alleviate myocardial hypertrophy, inflammation, oxidative stress and ischemia–reperfusion injury
Huangqi injection [116]	AMPK SIRT1 PGC-1α	ATP ROS NAD^+^	Restore SIRT-related deacetylation through mitochondrial protection	Improve mitochondrial function, reduce inflammatory injury, attenuate left ventricular remodeling

## 4. Challenges, Limitations, and Future Directions

### 4.1. Challenges and Limitations

The theoretical framework of TCM in the treatment of CHF through regulation of the metabolic–epigenetic axis is continuously improving and shows broad prospects. This field is currently at a critical bottleneck stage in the transition from hypothesis-driven research to empirical science, and a complete research framework spanning “association discovery,” “causal validation,” “systematic integration,” and “clinical translation” has not yet been established [117]. At present, most studies primarily focus on describing correlations between changes in metabolite levels, epigenetic modifications, and improvements in cardiac function [118]. Specifically, resveratrol intervention has been shown to ameliorate cardiac hypertrophy, myocardial fibrosis, mitochondrial dysfunction, inflammatory responses, and heart failure–related cardiac remodeling [75]. At the same time, natural product interventions can modulate key metabolic and epigenetic regulatory nodes, including SIRT1, AMPK, PGC-1α, Nrf2, PINK1/Parkin, and Smad3 acetylation, accompanied by improvements in cardiac structure and function [104]. However, there is still insufficient evidence to support a clear causal relationship between these changes. In other words, it remains unclear whether alterations in metabolite levels or metabolic enzyme activity are merely concomitant phenomena during disease improvement, or whether they directly drive cardioprotection through regulation of epigenetic modifications at specific genomic sites, and this has not yet been systematically elucidated [119,120]. While the causal mechanism remains to be clarified, the complexity of the material basis underlying the effects of TCM further increases the difficulty of mechanistic analysis [121]. Many natural products exhibit limitations such as low bioavailability, poor absorption, rapid in vivo metabolism, or dependence on gut microbiota transformation, leading to significant discrepancies between drug concentration, exposure duration, and chemical composition in in vitro experiments and the actual in vivo exposure conditions [122,123,124]. For example, flavonoids have been shown to regulate SIRT1, AMPK, mitophagy, inflammation, and fibrosis in cellular and animal experiments; however, their in vivo metabolic transformation, tissue exposure levels, and true active effectors remain to be further clarified [125,126]. Therefore, without support from pharmacokinetic, pharmacodynamic, and tissue distribution studies, extrapolation of in vivo mechanisms based solely on in vitro results may lead to a disconnect between “mechanistic interpretation” and the “true pharmacological effect” [127,128]. CHF is not caused by a single cellular abnormality or a single signaling pathway but rather represents a complex syndrome involving myocardial energy metabolism disorder, fibroblast activation, endothelial dysfunction, immune-inflammatory responses, neurohumoral dysregulation, and multi-organ interaction imbalance [129,130]. In particular, heart failure with preserved ejection fraction is closely associated with obesity, diabetes, hypertension, aging, and chronic inflammation. At present, many studies still rely on rodent models induced by a single stimulus or simplified in vitro cell systems, which fail to fully recapitulate the long-term progression and multifactorial nature of human chronic heart failure [131]. Although cellular models are useful for elucidating specific molecular mechanisms, they lack systemic regulatory context, including neuroendocrine, immune, vascular microenvironment, and inter-organ metabolic communication. Likewise, traditional animal models can capture structural and functional cardiac changes, but often fail to reproduce the complex comorbidity profiles observed in clinical patients [132]. In addition, conventional whole myocardial tissue homogenate-based methods provide only averaged signals, which may mask cell-type-specific alterations. This limitation also makes it difficult to explain how traditional Chinese medicine can simultaneously modulate multiple pathological processes, including inflammation, fibrosis, energy metabolism, and vascular function [133]. From a clinical translation perspective, there is still a lack of stable and reliable metabolic–epigenetic biomarkers, predictive indicators of therapeutic efficacy, and standardized evaluation systems. Moreover, significant heterogeneity exists in metabolic and epigenetic profiles across different etiologies, disease subtypes, and stages of heart failure, which further limits the translation of mechanistic findings into precision medicine applications [134].

### 4.2. Future Directions

Future research should shift from simply observing that metabolic changes occur concurrently with epigenetic alterations to systematically verifying whether specific metabolites directly regulate defined epigenetic enzymes or chromatin regions, ultimately determining cardiomyocyte fate and cardiac functional phenotypes [135,136]. To achieve this, CRISPR/dCas9-based epigenetic editing, targeted knockout or overexpression of key metabolic enzymes, metabolite supplementation or depletion strategies, isotope tracing, and chromatin immunoprecipitation sequencing [ChIP-seq] can be employed to construct a causal evidence framework linking “metabolites–epigenetic modifications–target gene expression–pathological phenotype,” thereby advancing the field from correlation-based description to mechanistic proof [137,138]. In parallel, greater emphasis should be placed on the in vivo dynamic characterization of active components of TCM and their metabolites, integrating pharmacokinetic parameters such as blood concentration, tissue distribution, target engagement, and pharmacodynamic responses to clarify the bioactive material basis underlying regulation of the metabolic–epigenetic axis, while also considering the role of gut microbiota in biotransformation and host metabolic remodeling [139]. Furthermore, research should progress from bulk tissue-level analysis toward high-resolution cellular and spatial profiling, given the marked heterogeneity of cardiomyocytes, fibroblasts, endothelial cells, smooth muscle cells, and immune cells in metabolic state, epigenetic landscapes, and responses to TCM interventions [140,141,142,143,144]. Accordingly, single-cell transcriptomics, single-cell epigenomics, spatial transcriptomics, spatial metabolomics, and integrated multi-omics approaches should be widely applied to delineate dynamic metabolic–epigenetic networks across distinct cell populations and spatial microenvironments, and to identify the key cell types, anatomical regions, and regulatory nodes targeted by TCM interventions [145,146,147]. Finally, the integration of artificial intelligence and network pharmacology may facilitate system-level modeling of the multi-component, multi-target, and multi-pathway characteristics of TCM, enabling a shift from single-target interpretation toward a network-based understanding of disease state remodeling [148,149]. This integrated approach will not only deepen the mechanistic understanding of TCM in heart failure but also support its international development and may ultimately enable a new therapeutic paradigm that systematically reverses disease-associated molecular memory through multi-target network modulation. Achieving this goal will require sustained interdisciplinary collaboration and continuous methodological innovation to address fundamental challenges in complex disease research.

## 5. Conclusions

CHF is a complex clinical syndrome characterized by multifactorial metabolic disturbances and persistent pathological remodeling. Emerging evidence indicates that myocardial metabolic reprogramming is not merely a consequence of energy imbalance but a central driver of epigenetic remodeling. Key metabolites, including α-KG, acetyl-CoA, NAD^+^, S-adenosylmethionine, succinate, and 2-HG, function as critical regulators of epigenetic enzymes and thereby link metabolic status to chromatin dynamics and gene transcription. Through this metabolic–epigenetic coupling, transient metabolic stress can be translated into stable pathological gene expression programs, contributing to cardiomyocyte hypertrophy, fibrosis, inflammation, apoptosis, and mitochondrial dysfunction. TCM, including active compounds, formula preparations, patent medicines, and injections, has demonstrated multi-level regulatory potential in CHF. By improving mitochondrial function, restoring TCA activity, and modulating key signaling pathways such as SIRT1, AMPK, PGC-1α, and Nrf2, TCM interventions may rebalance metabolite homeostasis and indirectly regulate epigenetic modifications, including DNA methylation and histone acetylation/methylation. These effects provide a mechanistic basis for its holistic and multi-target therapeutic characteristics. However, current evidence is still largely derived from preclinical models, and direct causal links between metabolic regulation, epigenetic remodeling, and functional cardiac improvement remain insufficiently validated. Future progress in this field will rely on integrated multi-omics approaches, including metabolomics, epigenomics, transcriptomics, and proteomics, combined with single-cell and spatial technologies to resolve cellular heterogeneity in the failing heart. In addition, CRISPR-based epigenetic editing, isotope tracing, and advanced pharmacokinetic–pharmacodynamic analyses will be essential to establish causal relationships within the metabolic–epigenetic axis. The integration of systems biology, network pharmacology, and artificial intelligence may further facilitate the transition from descriptive association studies to mechanistically grounded and clinically translatable models. In conclusion, the metabolic–epigenetic axis provides a unifying framework for understanding the pathogenesis of CHF and the therapeutic actions of TCM. Clarifying this regulatory network will not only deepen mechanistic insights into cardiac remodeling but also support the development of more precise and effective therapeutic strategies for heart failure.

## Figures and Tables

**Table 1:** Summary of the core effects and mechanisms of traditional Chinese medicine compounds and patent Chinese medicine in improving chronic heart failure by regulating the metabolic–epigenetic axis.

Name	Pathway	Metabolites	Epigenetic Effects	Improved Heart Function
QLQX [94]	AMPK SIRT1 PGC-1α TGF-β	NAD+ α-KG succinate	Activation of SIRT1, protection of TET/JmjC-KDMs activity	Reduce myocardial fibrosis and edema
Shexiang Bao Xin pill [95]	S1PR1 AKT STAT3	ROS	Reduction of inflammatory factor-mediated epigenetic abnormalities	Alleviate oxidative stress and myocardial injury
Shexiang Tongxin Dropping Pills [96]	ERK MAPK TGF-β Smad3	lactic acid	Reduction of abnormal histone Modification-mediated myocardial fibrosis	Relieve myocardial hypertrophy, and improve ventricular diastolic function
Yangxin Tablets [97]	PI3K AMPK PGC1α GLUT4	ATP	Activate the transcriptional activity of HIF-1α	Reduce myocardial oxygen consumption, decrease infarction area
Qidanlixin tablets [98]	Mtor p70S6k	IL-1β IL-6 TNF-α	Inhibit mTOR phosphorylation	Inhibit ventricular remodeling, and alleviate myocardial inflammatory infiltration
Xinshuitong capsule [99]	AQP1 AQP4 AQP7	ROS	Indirect suppression of inflammatory factor-mediated epigenetic abnormalities	Reduce myocardial edema and remodeling, reduce CHF mortality
Psychological prescription [100]	AGTR1 NLRP3	IL-1β IL-18 TNF-α	Inhibition of NLRP3 activation	Alleviate myocardial fibrosis and edema
Yi Xin Tai [88]	TMAO PKCNF-κB	TMAO IL-1β IL-6	Inhibiting the expression of NF-κB/PKC	Alleviate myocardial hypertrophy and fibrosis,
Kidney-invigorating soup [101]	p38MAPKp65NF-κB AQP4	L-leucine, β-carotene	Downregulation of the overexpression of pro-fibrotic genes	Improve myocardial ultrastructure and delay ventricular remodeling
Huangqi decoction [102]	NF-κB	ROS IL-1β IL-6 TNF-α	Downregulation of the overexpression of pro-inflammatory and apoptotic genes	Improve myocardial contractile function
Jin Xinkang [103]	CaN Drp1 Ca	ROS, Taurine Glycerophospholipids	Inhibit CaN/DRP1-mediated fetal gene overexpression	Decrease myocardial hypertrophy and fibrosis, reverse ventricular remodeling
Shengmai Yin [104]	Linoleic acid NF-κB	Regulate 14 biomarkers, such as LysoPC	Down-regulating the gene and protein expression of ALOX15 and CYP1A2	Decrease myocardial fibrosis and improve ventricular remodeling

## Data Availability

No new data were created or analyzed in this study. Data sharing is not applicable to this article.
